# The Effect of Maternal Postpartum Practices on Infant Gut Microbiota: A Chinese Cohort Study

**DOI:** 10.3390/microorganisms7110511

**Published:** 2019-10-30

**Authors:** Ying Wang, Yanqun Liu, Jinbing Bai, Xiaoli Chen

**Affiliations:** 1 Affiliation Wuhan University School of Health Sciences, Wuhan University, 169 Donghu Road, Wuhan 430071, China; wangying9398@163.com (Y.W.); liuyanqun1984@163.com (Y.L.); 2 Affiliation Emory University Nell Hodgson Woodruff School of Nursing, 1520 Clifton Road, Atlanta, GA 30322, USA; jinbing.bai@emory.edu

**Keywords:** infant, Chinese women, culture, probiotics consumption, microbiota, postpartum practices

## Abstract

(1) Background: The human gut microbiota at early life is shaped by numerous factors, especially factors from mothers, which have huge influence on infants’ gut microbiotas. The aim of this study was to investigate the effect of maternal adherence to Chinese traditional postpartum practices of “doing the month” on the development of infant gut microbiota at 6-month postpartum. (2) Methods: A cohort of 62 Chinese women at late pregnancy was recruited from a tertiary general hospital in a central region of China. The participants and their babies were followed up to 6 months postpartum. Finally, 50 mother-infant dyads were enrolled in the study. Women’s adherence to the traditional postpartum practices was measured by adherence to doing the month practices (ADP). Infant fecal samples were collected at six months of age and were analyzed using 16S rRNA V3 and V4 gene region sequences. (3) Results: *Ruminococcus gnavus* was significantly less abundant in infants whose mothers had a better adherence to the traditional postpartum practices of “doing the month.” Infants receiving *Clostridium-butyricum* during the first month after delivery had a significant dominance of *Escherichia/Shigella*. (4) Conclusions: Adherence to the traditional postpartum practices of “doing the month” can impact an infant’s gut microbiota at 6 months of age. Infants receiving probiotics during the first month after delivery had a significant dominance of opportunistic pathogens.

## 1. Introduction

The gut microbiota is the second most important genome of the human body. It represents a large microbial ecosystem which hosts trillions of microbial cells [1]. The gut microbiota provides essential functionality for the human body, including biosynthesis, metabolism, maturation and homeostasis of the human immune system, host cell proliferation, and vascularization and neurologic signaling [2]. A dysbiotic gut microbiota is associated with a variety of diseases and chronic conditions, such as diabetes [3], obesity [4], and allergenic diseases [5]. 

Recent research has uncovered the importance of the gut microbiota in human health and has attempted to understand the gut microbiota from the beginning of life. The ecological theory revealed that the priority effects (e.g., the order and timing of gut microbiota arrival) and microbial transmission (e.g., in utero transmission, infant delivery, and feeding modalities) determine subsequent development and health in later life [6,7]. Studies have reported the existence of a mother-to-infant gut microbiota transformation during the neonatal period [8,9,10]. A fetus acquires its microbiota in utero from its contact with maternal amniotic fluid [11], placenta [12,13], and umbilical cord blood [14]. After birth, the mother is still the main source of the infant’s gut microbiota, which includes maternal breast milk, skin, and the oral cavity [15]. Thus, various maternal factors can impact the development of the infant’s gut microbiota. Increasing evidence has demonstrated that maternal pre-pregnancy body mass index (BMI) and gestational weight gain (GWG) have direct impacts on infants. Excessive GWG, being overweight, or obesity before pregnancy regulated by maternal gut microbiota increase the risk of fetal gut microbiota imbalance, perinatal mortality, obesity, and metabolic syndromes [16,17]. Maternal stress during pregnancy affects the mother’s, as well as the infant’s, gut microbiota and increases the risk of anxiety in offspring [18,19]. Mueller et al. [20] also reported that the infant’s gut microbiota was affected through maternal antibiotic exposure during pregnancy, leading to obesity later in the infant’s life. Furthermore, a dysbiotic colonization of infant gut microbiota can result in detrimental healthcare outcomes in infants, such as higher rates of asthma, allergy, and gastrointestinal issues in later life [21].

Most recently, researchers have started to investigate the effect of human lifestyles and behaviors on gut microbiota in preclinical and clinical studies, including the study of diet patterns, physical activity, drinking, and smoking. Multiple studies suggest that diet and other lifestyle behaviors, such as physical activity, are primary factors determining the development and composition of the human gut microbiota [22,23,24]. In China, during the first month after delivery, postpartum women routinely adhere to the traditional maternal postpartum practices called “doing the month,” which are believed to restore body harmony and health, and prevent future illness [25]. “Doing the month” includes a series of practical behaviors related to the maternal role, such as activity limitations, maintaining warmth, and prescriptions about the types of food to consume or avoid [26]. As a unique set of culture-based postpartum practical behaviors, “doing the month” has been confirmed to lead to a limited maternal diet, poor hygiene, postpartum depression, and other physical discomfort in modern life [26]. Studies have shown that maternal nutritional composition influences the nutritional properties of breast milk [27], which serves as the main resource of microbiota that seeds the infant gut, and this could alter the infant’s gut microbiota to a large extent [28]. In addition, by directly contacting mothers’ skin, poor maternal hygiene may influence the infant gut microbiota [28]. “Doing the month” also leads to changes in maternal psychological health (e.g., depression and anxiety) [29], and this could impact the infant’s gut microbiota’s composition [30] Nevertheless, the effect of adherence to the traditionally practical postpartum behaviors—“doing the month,” on the development of the infant’s gut microbiota is unknown. We hypothesize that a mother’s adherence to the traditionally practical postpartum behaviors of “doing the month” is a major factor that affects the development of the infant’s gut microbiota at 6 months after delivery. 

The goals of this study were to: (1) investigate the effect of maternal adherence to traditional Chinese “doing the month” practical behaviors on an infant’s gut microbiota at 6 months; and (2) examine the influence of early life events on the infant’s gut microbiota, including antibiotic exposure, consumption of probiotics, delivery mode, and feeding type. Understanding associations between traditionally maternal postpartum behaviors and the gut microbiota in infants would provide a new angle to promote healthy growth and development in infants.

## 2. Materials and Methods 

### 2.1. Study design, Setting, and Participants

This study used a prospective maternal-child cohort design. Sixty-two women during their late pregnancy were recruited from an obstetric outpatient clinic in a tertiary hospital of Wuhan University in Wuhan, Hubei Province, China. The study was conducted between 4 March 2017 and 12 April 2018. This study was approved by the Research Ethics Boards of Medical School at Wuhan University (3 March 2017, JKHL2017-03-03). Informed consent statements were signed by all participants.

All women were selected independent of genetic background. The inclusion criteria were (1) pregnant women who planned to deliver a baby in a tertiary hospital of Wuhan University; and (2) a Wuhan resident. Pregnant women with pregnancy complications, women receiving antibiotic treatment during pregnancy, or those with cognitive impairment were excluded. Finally, this study included 50 mother-infant dyads (12 families could not be reached by telephone or email at 6 months postpartum) and 50 fecal samples were collected at home based on the Human Microbiome Project (HMP) protocol [31]. After collection, the fecal samples were transferred in a cooler (+4 °C, 1.5 h on average) and taken to the laboratory where they were frozen at −80 °C before DNA extraction. A total of 50 fecal samples were provided for high quality Illumina sequencing. 

### 2.2. Variables and Measures

The Chinese adherence to doing the month practices (ADP) was used to assess mothers’ ADP behaviors during the one-month postpartum period [25,26]. The score used for assessment ranges from 0 to 108, and a higher score indicates a higher level of adherence. The content validity index was 0.95 and Cronbach’s α was 0.86 [32]. This questionnaire was sent to participants by WeChat software at one month postpartum and women completed the online questionnaire via WeChat software.

Maternal and infant demographic data, GWG, and pre-pregnancy BMI were collected in the hospital based on maternal self-report 24 hours after childbirth. Infant antibiotic exposure, probiotic consumption, mode of delivery, and feeding type were ascertained when collecting samples at participants’ homes, based on maternal self-report. Pre-pregnancy weight was categorized as underweight, normal weight, overweight, or obese using the Asian-Pacific Recommendations [33]. According to the recommended range of the Institute of Medicine (IOM) [34], GWG was categorized as adequate, excessive, or low based on the pre-pregnancy weight.

The newborn consumption of probiotics containing *Clostridium butyricum* (*C. butyricum*, at least 75X10000000CFU) was meant to facilitate defecation and prevent jaundice in early neonates (0–1 month after birth). In this study, 35 parents prophylactically gave probiotic products to their healthy newborns despite the absence of physical signs of jaundice; among these infants, none of them developed jaundice during the course of the study. The use of probiotics was collected from the infant’s medical card, with the participants’ permission.

Infant antibiotic exposure was based on maternal self-report at the time of infant fecal sample collection. The antibiotics included cephalosporin and penicillin, which were given with intravenous fluids or orally. There were 16 infants that had antibiotic exposure during the 6 months postpartum.

Infant feeding type and the ADP questionnaire were collected one month after birth, based on mothers’ self-reports. Feeding type was also collected at six months after birth, coinciding with fecal sample collection, based on mothers’ self-reports. We categorized breast milk feeding groups as breast-fed or mixed feeding; infants who were mainly fed formula mixed with little breast milk were categorized as the mixed feeding group. None of the infants were fed solid food when fecal samples were collected.

### 2.3. DNA Extraction and PCR Amplification

Microbial DNA was extracted from fecal samples using the E.Z.N.A.^®^ soil DNA Kit (Omega Bio-tek, Norcross, GA, USA) according to manufacturer’s protocols. The final DNA concentration and purification were determined by NanoDrop 2000 UV-vis spectrophotometer (Thermo Scientific, Wilmington, DE, USA), and DNA quality was checked by 1% agarose gel electrophoresis. The V3-V4 hypervariable regions of the bacterial *16S* rRNA gene were amplified with primers 338F (5′- ACTCCTACGGGAGGCAGCAG-3′) and 806R (5′-GGACTACHVGGGTWTCTAAT-3′) by thermocycler PCR system (GeneAmp 9700, ABI, Walthma, MA, USA). The PCR reactions were conducted using the following program: 3 min of denaturation at 95 °C, 27 cycles of 30 s at 95 °C, 30 s for annealing at 55 °C, and 45 s for elongation at 72 °C, and a final extension at 72 °C for 10 min. PCR reactions were performed in triplicate using a 20 μL mixture containing 4 μL of 5 × FastPfu Buffer, 2 μL of 2.5 mM dNTPs, 0.8 μL of each primer (5 μM), 0.4 μL of FastPfu Polymerase and 10 ng of template DNA. The resulting PCR products were extracted from a 2% agarose gel and further purified using the AxyPrep DNA Gel Extraction Kit (Axygen Biosciences, Union City, CA, USA) and quantified using QuantiFluor™-ST (Promega, Madison, WI, USA) according to the manufacturer’s protocol. 

### 2.4. Illumina MiSeq Sequencing 

Purified amplicons were pooled in equimolar quantities and paired-end sequenced (2 × 300) on an Illumina MiSeq platform (Illumina, San Diego, CA, USA) according to the standard protocols by Majorbio Bio-Pharm Technology Co. Ltd. (Shanghai, China). 

### 2.5. Processing of Sequencing Data

Raw 16S rRNA sequencing data were demultiplexed, quality-filtered by Trimmomatic, and merged by FLASH with the following criteria: (1) the reads were truncated at any site receiving an average quality score <20 over a 50 bp sliding window. (2) Primers were exactly matched, allowing two nucleotide mismatches, and reads containing ambiguous bases were removed. (3) Sequences whose overlaps were longer than 10 bp were merged according to their overlap sequence.

Operational taxonomic units (OTUs) were clustered with a 97% similarity cutoff using UPARSE (version 7.1 http://drive5.com/uparse/), and chimeric sequences were identified and removed using UCHIME. The taxonomy of each 16S rRNA gene sequence was analyzed by RDP Classifier algorithm (http://rdp.cme.msu.edu/) against the Silva (SSU123) 16S rRNA database using a confidence threshold of 70%.

### 2.6. Statistical Methods

In the analysis, Alpha-diversity metrics were used, including diversity (Shannon, Simpson), richness (Chao, ace, sobs), and evenness (heip). In this study, Shannon indexes on OTU level were analyzed using Mothur (Version 1.30.2; www.mothur.org/). A larger Shannon value indicates the higher community diversity. 

Principal coordinate analysis (PCoA) was conducted according to the Bray–Curtis distance matrix calculated using OTU information from each sample. Permutational multivariate analysis of variance (PERMANOVA) was conducted to analyze the explanative degree of different grouping factors on the sample difference at the OTU level using the Bray–Curtis distance matrix. In PERMANOVA, we controlled confounders, including mode of delivery, feeding type, probiotic consumption, antibiotic exposure, and adherence to doing the month behaviors. Beta diversity metrics were analyzed based on mode of delivery, feeding type, probiotics consumption, and antibiotic exposure.

To investigate associations between maternal and infant characteristics and infant gut microbiota compositions, the linear discriminant analysis (LDA) effect size (LEfSe) method was implemented to identify the distinguishing taxa from phylum to genus levels within groups (http://huttenhower.sph.harvard.edu/galaxy/). A taxonomic bar chart and cladograms were used to visualize the results.

Multivariate analysis of general liner model (GLM) was used to adjust for possible confounders and to analyze the impact of significant factors on the diversity and abundance of the infant’s gut microbiota. This model used the significant genera within groups from LEfSe to adjust confounders. Additionally, to evaluate the effect of maternal “doing the month” on infant gut microbiota, all genera were included if they represented at least 20% of the infants and they had a minimum relative abundance of 0.25%. Finally, 21 genera meeting the prevalence cutoffs were included in the bioinformatic analysis. The following maternal characteristics were also included in the analysis: adherence behaviors of “doing the month,” delivery mode (categorical data: natural delivery versus C-section), antibiotic exposure (categorical data: yes versus no), feeding type (categorical data: breast feeding versus mixed feeding), and probiotic consumption (categorical data: yes versus no). 

Data regarding the relative abundances of genera were centered log ratio (CLR) transformed before the analysis. The multiple hypothesis tests were adjusted using Benjamini and Hochberg false discovery rate (FDR), and an association below an FDR threshold of 0.05 was considered significant. 

## 3. Results

### 3.1. Characteristics of Sequencing Results

A total of 2,551,852 usable sequences and 664 OTUs were obtained from 50 infants’ samples using Illumina MiSeq sequencing (Table 1). From these, 1,075,250 high-quality sequences were selected, with an average of 21,505 sequences per sample. In total, 532 OTUs were delineated at a 97% similarity level in infants after data trimming and quality filtering to an equal sequencing depth (43,953 reads per sample). The pan analysis indicated that only a small number of new, shared phylotypes would be expected with additional sequencing. The values of Good’s coverage for observed OTUs was 99.6 ± 0.03% (mean ± SD).

### 3.2. Microbiota Taxonomy 

**Figure 1:**
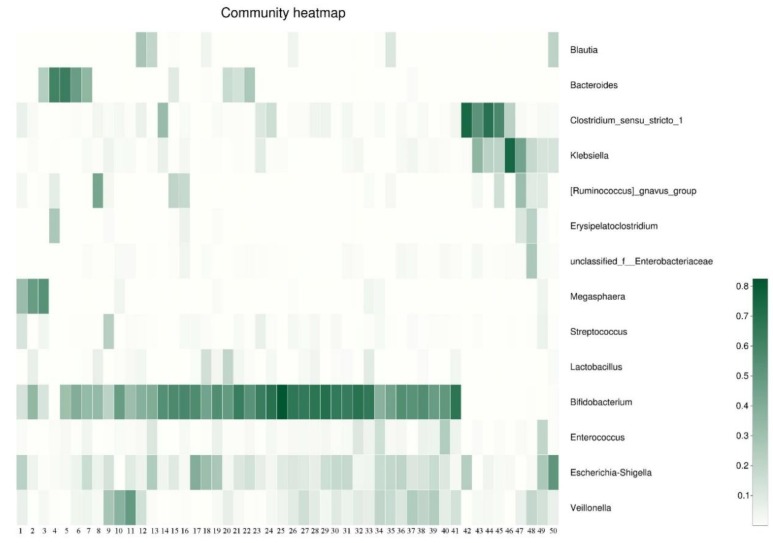
Relative abundance (≥1%) of infant gut microbiota at the genus level in the differentiation of the microbiota of six-month old infants. ** Each bar represents one sample.

The bacterial distribution was characterized in terms of the relative taxonomic abundances. A total of 21 phyla, 130 families, and 258 genera were found from 50 infants’ samples at 6 months postpartum. There were 54 genera present in at least 20% of infants’ gut microbiotas, and using 0.25% relative abundance as a cutoff, 21 genera met these conditions (Table S1). Figure 1 shows taxonomic compositions of the dominant bacteria on the genus level (relative abundance >1%). For the relative abundance, there were 14 genera above 1%, and the 10 most abundant genera were *Bifidobacterium* (38.18%), *Escherichia-Shigella* (10.63%), *Veillonella* (8.37%), *Clostridium sensu stricto* 1(7.64%), *Klebsiella* (5.37%), *Bacteroides* (5.3%), *Ruminococcus gnavus group* (3.29%), *Megasphaera* (3%), *Enterococcus* (2.88%), and *Lactobacillus* (1.72%).

### 3.3. Microbiotal Diversity

Based on the multivariate analysis of GLM, no significant factors were associated with Shannon’s index (mean ± SD: 1.81 ± 0.45) of infants’ gut microbiotas, controlling for maternal adherence to “doing the month,” feeding type, delivery mode, antibiotic exposure, and probiotic consumption.

**Figure 2:**
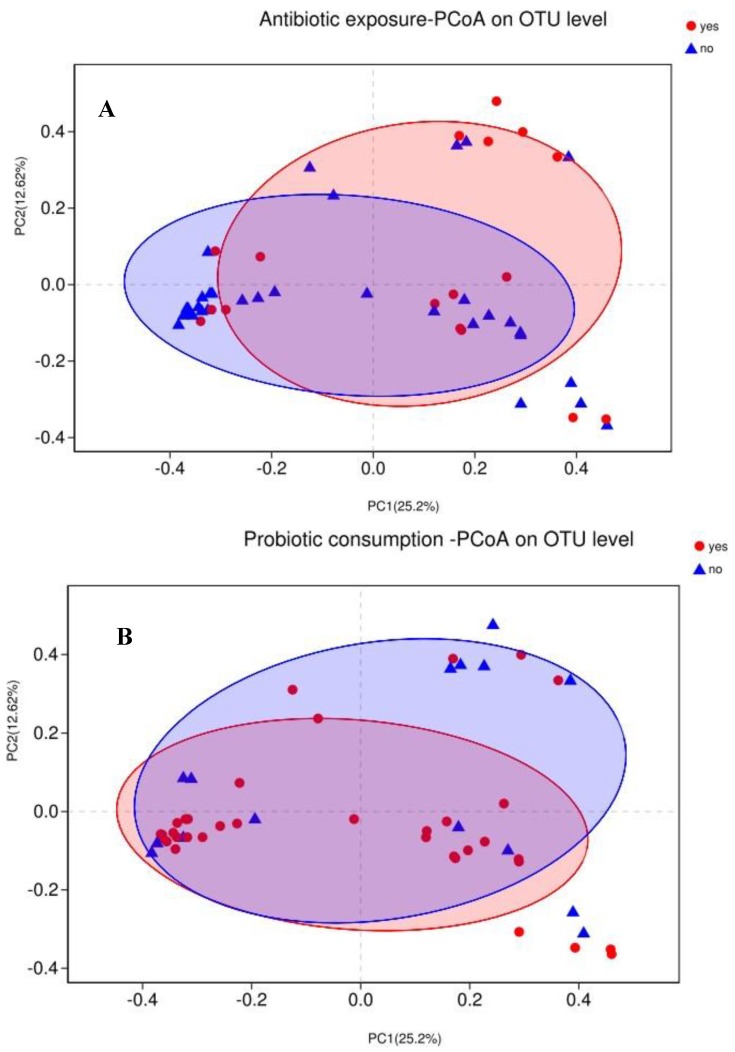

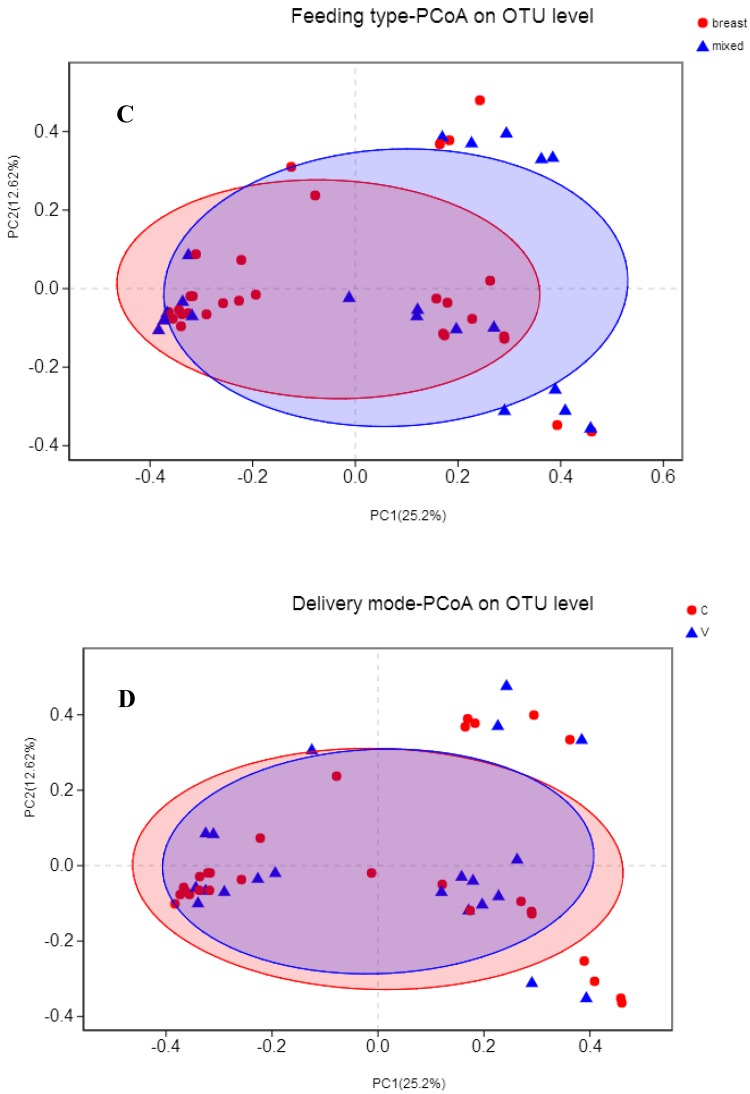
Principal coordinate analysis (PCoA) of infant gut microbiota using Bray–Curtis distance matrix. Two-dimensional PCoA was used to describe the relative abundance of infant gut microbiota. Each point represents a single sample and is colored by different groups (**A**) antibiotic exposure; (**B**) probiotic consumption; (**C**) feeding type; (**D**) delivery mode.

Variations in infants’ gut microbiota compositions among different groups (beta-diversity) were visualized by PCoA based on Bray–Curtis dissimilarity matrices on OTU level (Figure 2). Differences were further tested with PERMANOVA. Not surprisingly, the gut microbiota was not significantly separated based on antibiotic exposure (*R*^2^ = 0.03, adjust *p* = 0.24), probiotic consumption (*R*^2^ = 0.03, adjust *p* = 0.24), feeding type (*R*^2^ = 0.02, adjust *p* = 0.36), or delivery mode (*R*^2^ = 0.02, adjust *p* = 0.56).

### 3.4. Association between Maternal Adherence to “Doing the Month” and an Infant’s Gut Microbiota at 6 Months Postpartum

In the PERMANOVA analysis, there were no significant differences in maternal adherence to “doing the month” (*R*^2^ = 0.03, adjust *p* = 0.24). Controlling for confounders, we further explored the GLM by analyzing the impact of maternal adherence to “doing the month” on infants’ dominant genus and alpha diversity. According to the presence rate of no less than 20% and at least 0.25% relative abundance cutoffs, there were 21 genera included in the model. Results showed that *Ruminococcus gnavus* was statistically less abundant in infants whose mothers had higher adherence to the Chinese traditional practices of “doing the month” (*β* = −43.52, CI = (−72.9, −14.14), *p* = 0.005, adjust *p* = 0.012), and *Ruminococcus gnavus* was the seventh most abundant genus among infants. *Intestinibacter* (*β* = 0.02, CI = (0.001, 0.039), *p* = 0.035, adjust *p* = 0.376) and *Tyzzerella* (*β* = 0.019, CI = (0.0001, 0.037), *p* = 0.047, adjust *p* = 0.376) were positively correlated with maternal adherence to “doing the month.” However, after FDR adjusting, there were no significant relationships between maternal adherence to “doing the month” and an infant’s gut microbiota at 6 months postpartum. 

### 3.5. Association between Events in Early Life and an Infant’s Gut Microbiota at 6 Months Postpartum

**Figure 3:**
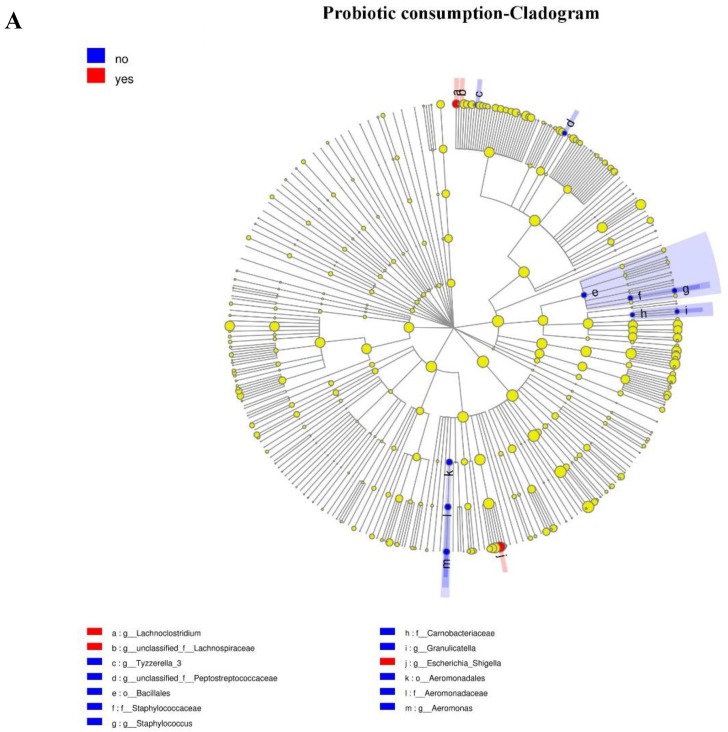

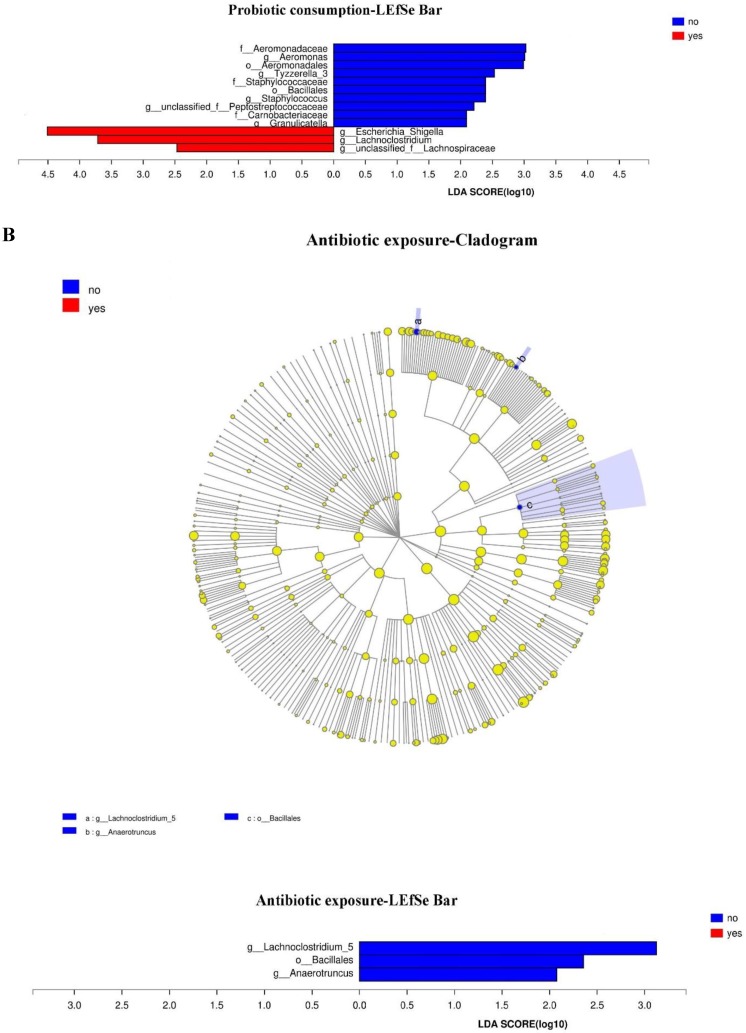

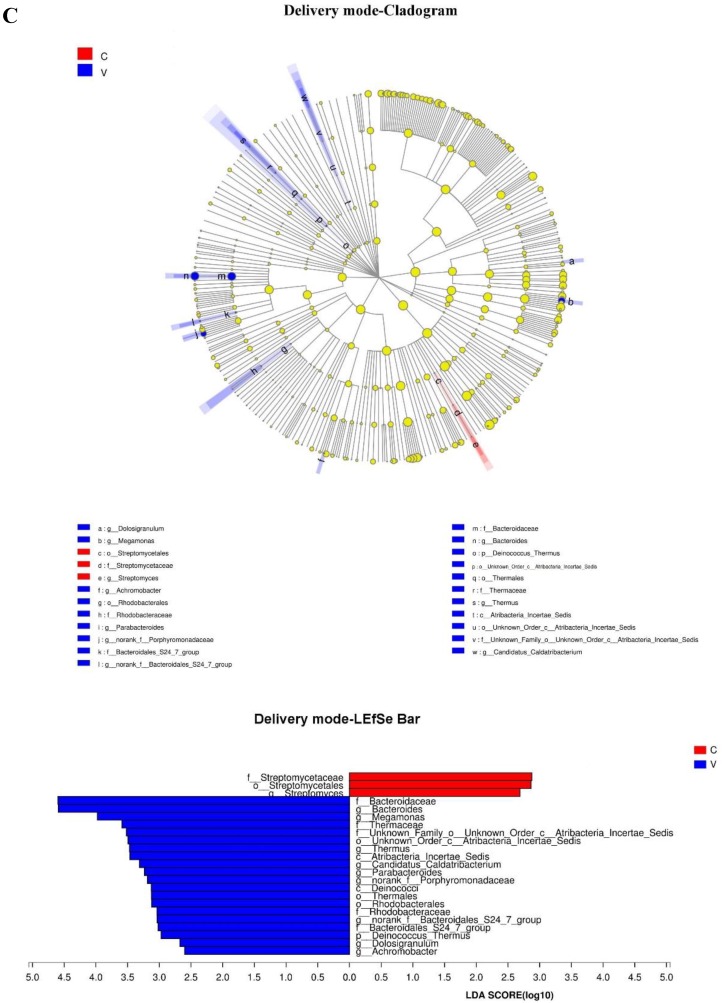

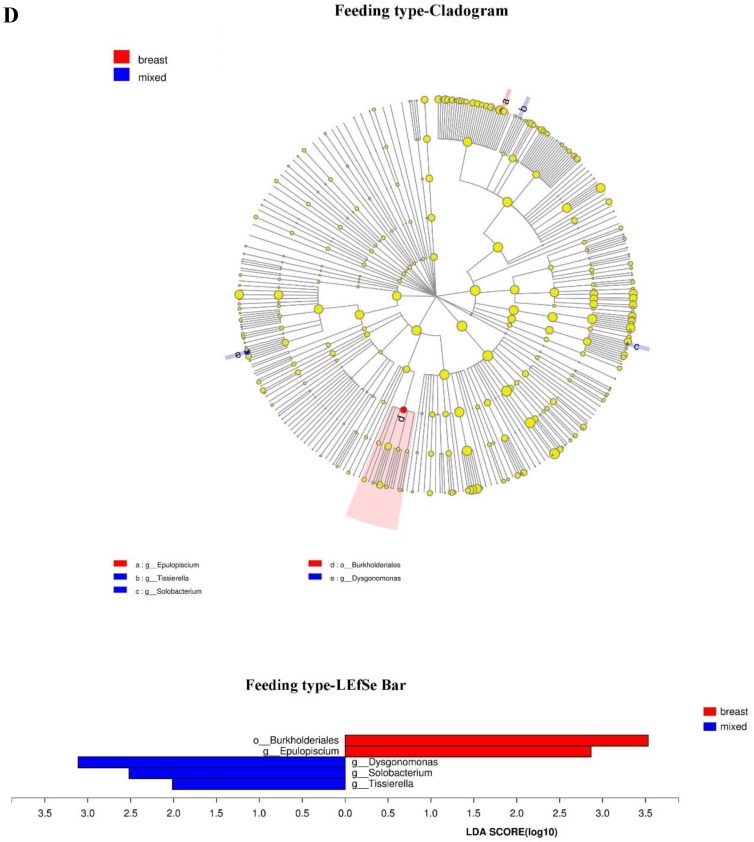
Taxonomic representation of statistically and biologically consistent differences between different groups ((**A**) probiotic consumption; (**B**) antibiotic exposure; (**C**) delivery mode; (**D**) feeding type). Differences are represented by the color of the most abundant genus. The diameter of each circle is proportional to taxon abundance; from inside to outside circle indicate the phylum to genus level in turns (g: genus; f: family; o: order; c: class). Histogram of the linear discriminant analysis (LDA) scores for differentially abundant genera. The threshold on the logarithmic LDA score for discriminative features was set to 2.0; the larger the LDA score, the greater influence of species abundance on the difference between two groups. ((**A**) Probiotic consumption: More abundant in no consumption group: *Aeromonadaceae*, *Aeromonas*, *Aeromonadales*, *Tyzzerella*, *Staphylococcaceae*, *Bacillales*, *Staphylococcus*, *Peptostreptococcaceae*, *Carnobacteriaceae*, and *Granulicatella.* More abundance in consumption group: *Escherichia_Shigella*, *Lachnoclostridium*, and *Lachnospiraceae*. (**B**) Antibiotic exposure: More abundance in no antibiotic exposure group: *Lachnoclostridium* and *Bacillales*, *Anaerotruncus*. (**C**) Delivery mode: More abundance in Cesarean section group: *Streptomycetaceae*, *Streptomycetales* and *Streptomyces*. More abundance in vaginal delivery group: *Bacteroidaceae*, *Bacteroides*, *Megamonas*, *Thermaceae*, *(f)Atribacteria Incertae Sedis*, *(o)Atribacteria Incertae Sedis Thermus*, *(c)Atribacteria Incertae Sedis*, *Candidatus Caldatribacterium*, *Parabacteroides*, *Porphyromonadaceae*, *Deinococci*, *Thermales*, *Rhodobacterales*, *Rhodobacteraceae*, *(g)Bacteroidales S247 group*, *(f)Bacteroidales S247 group*, *Deinococcus Thermus*, *Dolosigranulum*, and *Achromobacter*. (**D**) Feeding type: More abundance in breast-fed group: *Burkholderiales* and *Epulopiscium*. More abundance in mixed-fed group: *Dysgonomonas*, *Solobacterium*, and *Tissierella*).

Because of the absence of any clear clusters in PCoA for gut microbiota within groups, the LEfSe method was implemented to identify the distinguishing taxa from the phylum to genus level within groups including probiotic consumption, delivery mode, feeding type, and antibiotic exposure (Figure 3). Furthermore, the significantly different genera were incorporated into the GLM to control confounders. Results showed that infants who consumed probiotics had a dominance of *Escherichia-Shigella* (β = 287.44, CI = (172.79, 558.09), *p* = 0.038, adjust *p* = 0.042), as the second most abundant genus. There were no differences in genera abundance within delivery mode (natural delivery versus C-section), antibiotic exposure (yes versus no), or feeding type (breast versus mixed) groups.

## 4. Discussion

Recent evidence has shown that the acquisition and development of an infant’s microbiota are key to establishing a healthy host-microbiotal symbiosis. The maternal microbial reservoir plays a crucial role in this process [15,35,36]. In this study, we found that maternal adherence to traditionally practical behaviors of “doing the month” in the postpartum period was associated with a lower abundance of *Ruminococcus gnavus* genus in the infant’s gut microbiota. *Ruminococcus gnavus*, which has well defined roles in intestinal mucus degradation and immune function, increases the probability of infectious diarrhea [37]. Specifically, the *Lachnospiraceae* family, which includes the genus *Ruminococcus* spp., has been proven to be associated with protection against *C. difficile* infections [38] and obesity [39].

We hypothesized that maternal adherence to “doing the month” might influence the infant gut microbiota by three pathways. Firstly, women with higher maternal adherence to traditional practices of “doing the month” generally had poor personal hygiene and did not bathe or wash their hair during the one-month postpartum period [29]. A recent study suggested that 10.3% of infants’ gut microbiotas were received from mothers’ areolar skin during the first 30 days of life [28]. By directly contacting with mothers’ skin and surrounding environments, higher maternal adherence to traditional practices of “doing the month” might decrease *Ruminococcus* spp. in infants due to poor maternal hygiene. Secondly, infant gut microbiota could be affected through breast-feeding. During the first month postpartum, higher adherence to traditional practices of “doing the month” are always accompanied with higher probability of a single diet [26], sleeping problems [32], and decreased immunity [40], all of which could modulate maternal breast milk directly or indirectly by the hypothalamic–pituitary–adrenal (HPA) axis. Breast milk is the main resource seeding infants’ gut microbiotas [28], which could alter abundance of *Ruminococcus* spp. in infants’ gut microbiotas. In addition, during the first month after birth, postpartum women spend a large amount of daily time in bed. The restriction of physical activity is known to cause musculoskeletal and cardiovascular deconditioning [32,41]. For maternal psychological health, studies suggested that longer adherence to traditional practices of “doing the month” increased stress and postpartum depression [42,43]. Higher adherence to “doing the month” makes postpartum women prone to stress and depression [29], and maternal psychological health may affect an infant’s gut microbiota’s composition [30]. In a longitudinal study, Zijlmans et al. prospectively investigated the relationship between exposure to prenatal stress and infants’ gut microbiotas at 3 months of age; in their study, 58 women in their third trimester were assessed for prenatal stress, and their infants’ gut microbiotas were assessed at 3 months of age. They found that increasing abundance of *Proteobacteria*, as well as decreased lactic acid bacteria and *Bifidobacteria*, were associated with infants being born to mothers with high levels of reported stress. The study was the first to prove that maternal stress could modulate an infant’s gut microbiota [30].

In this study, results showed that infant gut microbiota did not show any difference in breast milk infants and mixed feeding infants. Evidence has shown that feeding type is a major factor determining early microbial colonization, influencing the neonatal gut microbiota composition and gastrointestinal function [34]. Studies have reported that the gut microbiotas of breast-fed infants contain higher levels of *Lactobacilli* and *Bifidobacteria* and lower levels of potential pathogens compared with those with formula feeding [44,45,46], which was inconsistent with our findings. A likely explanation was that the majority of infants in this study were breast-fed during the first month; however, most were formula-fed for the remainder of our study. Another study also confirmed that infants fed breast milk exclusively showed the same composition of gut microbiota compared with those receiving formula and breast milk [47].

We explored the effect of *Clostridium butyricum* consumption at early life on infant gut microbiota. Of the 35 healthy infants who were prophylactically given *Clostridium butyricum* to prevent jaundice, none developed jaundice over the course of this study; we found that these infants had a dominance of *Escherichia/Shigella* compared with those not receiving *Clostridium butyricum*. *Clostridium butyricum* is known as a butyrate producer and a regulator of gut health [48]. As an anaerobic, Gram-positive, butyric acid-producing bacillus, *Clostridium butyricum* resides in the gastrointestinal tract and has a protective role against pathogenic bacteria and intestinal injury by modulating gut microbial metabolites, such as short-chain fatty acids (SCFAs) [49]. Several studies have indicated that, via modulation of the gut microbiota, *Clostridium butyricum* effectively improved major depressive disorder [50], irritable bowel syndrome (IBS) [51], allergic rhinitis [52], and cerebral ischemia/reperfusion (I/R) injury [48]. They also suggested that *Clostridium butyricum* influenced microbial metabolism by optimizing the structure of microbiota and enhancing acetate production and utilization for butyrate production [53]. In our study, the oral administration of *Clostridium butyricum* during early life had an influence on infants’ gut microbiota’s compositions at 6 months of age. It significantly increased the Gram-negative, conditionally pathogenic *Escherichia/Shigella* at 6 months of age. Further clinical studies considering these factors should explore possible roles of *Clostridium butyricum*’s consumption in gut microbiol development. We should note that the overuse of probiotics in healthy infants is a serious problem that may pose a threat to human health. Studies in healthy adults have revealed that probiotic consumption cannot improve blood lipid profiles, and its benefits (e.g., stool consistency and bowel movement) are temporary [54]. Despite the current lack of warnings, there is insufficient evidence to conclude that probiotics have no deleterious side effects. The use of probiotics in healthy infants in order to provide sustained health benefits requires more in-depth study to confirm.

The delivery mode can affect infants’ gut microbiota [55,56,57]. Our study showed that differences caused by delivery mode were absent in the infants of six months. Chu et al. [58] proved that the gut microbiotas of neonates were influenced by mode of delivery at birth, but the influences disappeared at 6 weeks after birth. Compared with vaginally born infants, Penders et al. [59] found that those Cesarean-section born had lower abundance of *Bifidobacteria* and *Bacteroides*, whereas they had *Clostridium difficile* predominance. Another study showed that differences in infants’ early gut microbial colonization caused by delivery mode persisted 4 months after birth [55,60]. At 3 months after birth, the abundances of *Actinomycobacteria* and *Bacteroides* were relatively lower, and infants delivered by Cesarean section had a predominant *Firmicutes phylum*. For vaginally delivered infants, the dominant bacteria were *Bifidobacteria* and *Bacteroides*. *Clostridium* and *Lactobacillus* were the dominant bacteria for infants delivered by Cesarean section. However, these differences disappeared 6–12 months after delivery [61]. Given the complexities of the research results, our studies made an assumption: that diet consumption was an important factor to renovate gut microbiota in early human life [62]. The structures and compositions of infants’ gut microbiotas born with Cesarean section were similar to those of infants delivered vaginally at 4 months [60]. With the transmission of solid food, it is meaningful to investigate how dietary patterns influence an infant’s gut microbiota.

Recently, the public has been paying more attention to the abuse and overuse of antibiotics. In a 3-year longitudinal study of 39 infants in England, Yassour et al. found that there was a lower diversity of gut microbiota in antibiotic treated children than untreated children [63]. After antibiotic exposure one month later, the gut microbiota appeared to return to normal, which was consistent with our study. However, Bokulich et al. analyzed 43 infants over the first 2 years of life in the United States [57]. Infants who had prenatal and postnatal antibiotic treatment during the first 24 months of life showed a delayed development of the gut microbiome and suppressed *Clostridiales*, including *Lachnospiraceae*. However, considering the different durations, doses, ages, and approaches to antibiotics including the deviations between the methods of administration, it is not surprising that the results were incomparable among themselves. Numerous studies have shown that antibiotic exposure contributed to poor infant health at a later stage of life, such as by increasing the risk for childhood obesity [20], asthma [64], food allergies [65], and inflammatory bowel disease [66]. Future studies will need to keep exploring the impact of antibiotic use on children’s health and development longitudinally.

## 5. Limitations

Although our study attempted to provide a comprehensive look into the relationships between maternal postpartum practices, including other relevant factors (e.g., antibiotic exposure, probiotics consumption, delivery mode, and feeding type), and infant gut microbiota at 6 months of age, there are several limitations to be addressed in future studies. First, it would have been more significant if we had explored the effect of antibiotics on infants’ gut microbiotas in a quantificational measurement. Second, the study design of 50 cross-sectional samples at 6 months of age, rather than at earlier age, may have missed capturing effects of early maternal practices and factors (i.e., “doing the month,” mode of delivery, feeding type, antibiotic exposure, and probiotic consumption) on infants’ gut microbiotas. Third, it is important to collect longitudinal samples to understand the longitudinal dynamics of the gut microbiotas in infants and their influencing factors. Finally, the data on relative abundance were not sensitive enough to distinguish difference among groups compared to absolute quantification.

## 6. Conclusions

The results suggested that maternal adherence to traditionally practical behaviors of “doing the month” can induce changes in the gut microbiotas of infants. It emphasized the importance of mothers’ behaviors in seeding infants’ gut microbiotas. This study showed that *Clostridium butyricum* use early in life could increase conditional pathogenic bacteria *Escherichia/Shigella*. Probiotic use has raised enormous safety concerns. A longitudinal study with a larger sample size should be conducted.

## Figures and Tables

**Table 1:** Demographic and birth characteristics of infants (*n* = 50).

Items	Total *N* (%)/Mean (SD)
Maternal age	30.13 (27.01−33.25)
Gestation	
1	31 (62%)
2	12 (24%)
3	6 (12%)
4	1 (2%)
Parity	
0	34 (68%)
1 prior child	16 (32%)
Maternal education	
<12 a	2 (4%)
12 a	31 (62%)
>12 a	17 (34%)
Pre-pregnancy BMI category	
Underweight	9 (18%)
Normal	35 (70%)
Overweight	6 (12%)
GWG category	
less	8 (16%)
adequate	20 (40%)
excessive	22 (44%)
Gestational week	39.69 (38.73–40.65)
Mode of delivery	
Vaginal	22 (44%)
Cesarean section	28 (56%)
Adherence to “doing the month”	81.94 (66.34–97.54)
Feeding type	
Breast	30 (60%)
Mixed	20 (40%)
Probiotic consumption	
Yes	35 (70%)
No	15 (30%)
Antibiotic exposure	
Yes	17 (34%)
No	33 (66%)
Birth weight	3.45 (3.09–3.81)
Sex	
Male	20 (40%)
Female	30 (60%)

## Supplementary Materials

Supplementary materials can be found at https://www.mdpi.com/2076-2607/7/11/511/s1.
